# Taking Language out of the Equation: The Assessment of Basic Math Competence Without Language

**DOI:** 10.3389/fpsyg.2018.01076

**Published:** 2018-06-26

**Authors:** Max Greisen, Caroline Hornung, Tanja G. Baudson, Claire Muller, Romain Martin, Christine Schiltz

**Affiliations:** ^1^Cognitive Science and Assessment Institute, University of Luxembourg, Luxembourg, Luxembourg; ^2^Luxembourg Centre for Educational Testing, University of Luxembourg, Luxembourg, Luxembourg

**Keywords:** nonverbal, assessment, mathematics, language, dyscalculia, video, instruction, screener

## Abstract

While numerical skills are fundamental in modern societies, some estimated 5–7% of children suffer from mathematical learning difficulties (MLD) that need to be assessed early to ensure successful remediation. Universally employable diagnostic tools are yet lacking, as current test batteries for basic mathematics assessment are based on verbal instructions. However, prior research has shown that performance in mathematics assessment is often dependent on the testee's proficiency in the language of instruction which might lead to unfair bias in test scores. Furthermore, language-dependent assessment tools produce results that are not easily comparable across countries. Here we present results of a study that aims to develop tasks allowing to test for basic math competence without relying on verbal instructions or task content. We implemented video and animation-based task instructions on touchscreen devices that require no verbal explanation. We administered these experimental tasks to two samples of children attending the first grade of primary school. One group completed the tasks with verbal instructions while another group received video instructions showing a person successfully completing the task. We assessed task comprehension and usability aspects both directly and indirectly. Our results suggest that the non-verbal instructions were generally well understood as the absence of explicit verbal instructions did not influence task performance. Thus we found that it is possible to assess basic math competence without verbal instructions. It also appeared that in some cases a single word in a verbal instruction can lead to the failure of a task that is successfully completed with non-verbal instruction. However, special care must be taken during task design because on rare occasions non-verbal video instructions fail to convey task instructions as clearly as spoken language and thus the latter do not provide a panacea to non-verbal assessment. Nevertheless, our findings provide an encouraging proof of concept for the further development of non-verbal assessment tools for basic math competence.

## Introduction

Basic counting and arithmetic skills are necessary to manage many aspects of life. Although primary education focuses on these subjects, 5–7% of the general population suffer from mathematical learning difficulties (MLD) (Butterworth et al., 2011), often leading to dependence on other people or technology.

Early diagnostic is key to remedying MLD (Gersten et al., 2005). Basic mathematical skills, e.g., counting, quantity comparison, ordering, and simple arithmetic are the strongest domain-specific predictors for mathematical performance in later life (Desoete et al., 2009; Jordan et al., 2010; LeFevre et al., 2010; Hornung et al., 2014). Valid MLD assessments exist in various forms and for all ages (van Luit et al., 2001; Haffner et al., 2005; Schaupp et al., 2007; Noël et al., 2008; Aster et al., 2009; Ricken et al., 2011). However, all of them rely on verbal instructions and (in part) verbal tasks.

This is a problem. First, performance in mathematical tests is predicted by the pupils' proficiency in the instruction language (Abedi and Lord, 2001; Hickendorff, 2013; Paetsch et al., 2016). Others have shown that the complexity of mathematical language content of items is predictive of performance (Haag et al., 2013; Purpura and Reid, 2016). Diagnostic tools for MLD relying on language may therefore significantly bias performance in test-takers that are not proficient in the test language, leading to invalid results (see Scarr-Salapatek, 1971; Ortiz and Dynda, 2005 for similar considerations concerning intelligence testing). Furthermore, the match between math learners' language profiles and the linguistic context in which mathematical learning takes place plays a critical role in the acquisition and use of basic number knowledge. Matching language contexts improve bilinguals' arithmetic performance in their second language (Van Rinsveld et al., 2016), and neural activation patterns of bilinguals solving additions differ depending on the language they used, suggesting different problem-solving processes (Van Rinsveld et al., 2017).

In linguistically homogeneous societies, where the mother tongue of most primary school children matches the language of instruction and assessment tools, this is less of a problem. It is however critical in societies with high immigration and, therefore, linguistically diverse primary school populations. In Luxembourg, for instance, where the present project is located, currently 62% of the primary school students are not native Luxembourgish speakers (Ministère de l'éducation nationale de l'enfance et de la Jeunesse, 2015). Due to migration, multilingual classrooms are steadily becoming the rule rather than the exception (e.g., from 42% foreign speakers in 2004 to 62% in 2014) (Ministère de l'éducation nationale de l'enfance et de la Jeunesse, 2015), likely increasing the urgency of the problem in the future.

Even in traditionally multilingual contexts, diagnostic tools for the assessment of basic numerical abilities in early childhood are available in a few selected languages only, usually those that are best understood by most, yet not necessarily all students. As described above, this leads to invalid conclusions about non-native speakers' ability. In addition, comparisons between different tools and even different linguistic versions of the same tool are difficult because the norms they are based on are usually collected in linguistically homogenous populations and can thus not be extrapolated to populations with different linguistic profiles.

The present study originated in a project that aims to develop a test of basic numerical competencies which circumvents linguistic interference by relying on non-verbal instructions and task content. In the field of intelligence assessment, the acknowledgment of language interference has led to the development of numerous non-verbal test batteries (Cattell and Cattell, 1973; Lohman and Hagen, 2001; Naglieri, 2003; Feis, 2010). However, these tools tackle only the problem of verbal tasks, not of verbal instructions. The same is true for numeracy assessment. Although many test batteries (e.g., Tedi-MATH, Zareki-R, ERT0+, OTZ, Marko-D, to name a few) use non-verbal and non-symbolic tasks (e.g., arithmetic, counting, or logical operations on numbers), they still rely on verbal instructions, which may limit the testee's access to the content. Linguistic simplification of mathematics items can improve performance for language minority students (Haag et al., 2014). However, we think that for many simple tasks, verbal content and instructions can be avoided altogether. These tasks that children of (above-) average ability usually solve easily are crucial to the diagnosis of MLD, as they allow for a differentiation of children's numerical abilities at the bottom end of the ability distribution. Hence, non-verbal assessment of basic mathematical skills may help identify children in need of intervention at an early age and independently of their linguistic abilities, thus reducing the bias that common assessments often suffer from. Comparable approaches have been taken in the field of intelligence testing for the hearing-impaired, in which pantomime instructions for the Wechsler performance scale have been explored (Courtney et al., 1984; Braden and Hannah, 1998).

With this goal in mind, using available test batteries and the official study plan (MENFP, 2011) as a reference for task content and design, we developed different task types for which a valid non-verbal computerized implementation was possible. Governmental learning goals for preschool mathematics include but are not limited to: Ability to represent numbers with concrete material, ordering abilities (range 0–10), definition, resolution & interpretation of an arithmetical (addition/subtraction) problem based on images and mental addition/subtraction (range 0–10).

The tasks we developed encompass and measure all the above competencies: Quantity representation, ordering abilities as well as symbolic and non-symbolic arithmetic. We chose to add a quantity comparison task as it has been found to be one of the most consistent predictors of later math performance (e.g., De Smedt et al., 2009; Sasanguie et al., 2012; Nosworthy et al., 2013; Brankaer et al., 2017; see Schneider et al., 2017 for a meta-analysis). Instead of using verbal instructions, we convey task requirements with the use of videos that show successful task completion and interactions with the tasks from a first-person point of view. Prior research has shown improved performance in a computerized number-line estimation task for participants who viewed videos of a model participant's eye gaze or mouse movements, compared to control conditions both with and without anchor points (Gallagher-Mitchell et al., 2017).

The aims of the present study were to evaluate whether basic math competence can be assessed on a tablet PC without language instructions and whether the mode of instruction affects performance. To this end, we designed a set of computerized tasks based on validated assessments measuring basic non-symbolic and symbolic mathematical abilities, which were administered either non-verbally (using computer-based demonstrations; experimental condition) or traditionally (using verbal instructions; control condition). Because young school children's attention span is limited (Pellegrini and Bohn, 2005), some of the tasks were administrated to one sample (Sample 1) in a first study and the remainder to another sample (Sample 2) in a second study 5 months later. First, considering that the non-verbal mode of instruction was new, we examined possible difficulties both directly (understanding of feedback and navigation) and indirectly (repeated practice sessions). Second, though tasks were derived from field-tested assessments, performance on the new tasks was correlated with performance on two standardized and one self-developed measure in order to ensure task validity. Third, we examined students' performance compared by condition and overall. Considering the novelty of the non-verbal task administration, we did not specify directed hypotheses but examined this question exploratively.

## Methods

### Participants

Table 1 shows participant demographics, language background and socio-economic status. The ISEI is the International Socio-Economic Index of Occupational Status, used in large scale assessments. It ranges from 16 (e.g., agricultural worker) to 90 (e.g., judge). An average ISEI of 50 will thus indicate above average socio-economic status. As we could not directly assess socio-economic status in our studies, ISEI was estimated based on the communes in which the studies took place. This data is publicly available and in Luxembourg the communes average ISEI ranges from 35 to 65. All participants were recruited from first grade in Luxembourg's primary schools with the authorization of the Ministry of Education and the directors of the participating school sectors. Participants from the first sample were tested after 5 weeks of schooling while participants from the second sample were tested after 28 weeks of schooling. Teachers interested to participate in the study with their classes received information and consent letters for the pupil's legal representatives. Only pupils whose parents consent was obtained participated in this study. All children in Luxembourg spend two obligatory years in preschool and about a third of them participate in an optional third year of preschool prior to the two mandatory years (Lenz, 2015).

**Table 1:** Participant demographics, language background and SES.

**Sample**	***N***	**% girls**	**Age**	**Schooling**	**Language**			**ISEI**
			***M* (*SD*)**		**% RO**	**% LG**	**% OT**	***M* (*SD*)**
Sample 1	96	53.1	6 years; 7 months (4 months)	Grade 1 (5 weeks)	30.2	55.2	14.6	50 (6.3)
Sample 2	141	48.2	7 years; 2 months (4 months)	Grade 1 (28 weeks)	55.3	34.8	9.9	47.9 (7.2)

*% RO, percentage of Romance language speaking children (French, Portuguese, Italian, Spanish). % LG, percentage of children speaking Luxembourgish or German. % OT, percentage of children with other language backgrounds (Slavic, English). ISEI, International Socio-Economic Index of Occupational Status*.

### Materials

#### Experimental tasks

**Figure 1:**
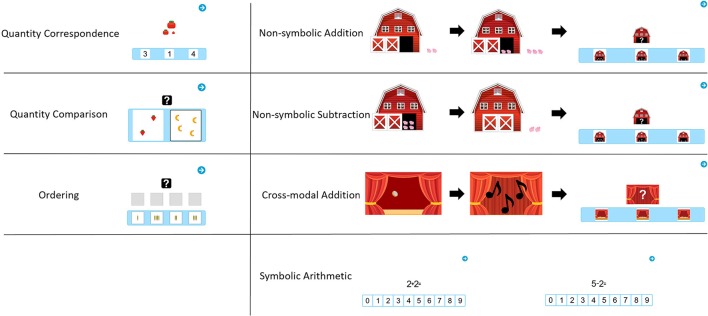
Example Images of the experimental tasks.

As mentioned, the two samples received different types of tasks. In the following, all task types will be described in order of their administration. The number in parentheses after each task name indicates the sample it was administered to. Example images for each task are presented in Figure 1.

#### Quantity correspondence (S1)

The first task required determination of the exact quantity of the target display and choosing the response display with the corresponding quantity (both ranging from 1 to 9). Each item consisted of a target quantity displayed at the center of the screen (stimulus). The nature of the quantity was varied and was either non-symbolic (based on real objects [fruit], abstract [dot collections]) or symbolic (Arab numerals). In the lower part of the screen, three different quantities were displayed to the participant from which he/she was to choose the one corresponding to the stimulus (multiple-choice images). The item pool consisted of five subgroups of items containing four items each:

1. Non-symbolic, *identical* objects for stimulus and multiple-choice images
2. Non-symbolic, *different* objects for stimulus and multiple-choice images
3. Non-symbolic, collections of black dots of variable sizes and configurations
4. Symbolic, Arabic numerals in both stimulus and multiple-choice images
5. Mixed (combinations of the preceding characteristics)

Image characteristics (object area, total occupied area, etc.) were manually randomized but not systematically controlled for.

#### Quantity comparison (S1)

The second task required determining and choosing the larger of two quantities (range: 1–9) displayed at the center of the screen. The nature of the quantities was varied similarly to the first task:

1. Non-symbolic, each quantity being composed of different objects (4 items)
2. Non-symbolic, each quantity being composed of collections of black dots of variable sizes and configurations (4 items)
3. Symbolic, at least one of the two displays showing an Arabic numeral (4 items)

#### Ordering (S1)

The third task required reordering 4 images by increasing quantities (range 1–9). The characteristics were divided into 2 subgroups, represented by 4 items each:

1. Ordering based on non-symbolic quantity
2. Ordering based on numerical symbols (Arabic digits)

#### Non-symbolic addition (S2)

The first task required to solve a non-symbolic addition problem. Participants saw an animation of 1–5 pigs entering a barn. The barn door closed. Then, the door opened again, and 1–5 more pigs entered the barn. The door closed again. The result range included the numbers from 3 to 8 only. In the **non-symbolic answer** version of this task (3 items), participants were then presented with three images containing an open barn with pigs inside. Their task was to choose the image showing the total number of pigs left in the barn. In the **symbolic answer** version of the task (3 items), participants selected the correct number of pigs from an array of numerals from 0 to 9 in ascending order to choose from.

#### Non-symbolic subtraction (S2)

The second task required solving a non-symbolic subtraction problem using the same pigs-and-barn setting described above. Participants were shown an animation of an open barn containing some pigs, after which some pigs left and the barn door closed. The minimum number of pigs displayed in a group was 2, the maximum was 9. The result range was from 1 to 6. **Symbolic** and **non-symbolic** answer versions (3 items each) were the same as above.

#### Crossmodal addition (S2)

The third task for Sample 2 required solving a crossmodal addition problem using visual and auditory stimuli. Participants saw an animation of coins dropping on the floor, each one making a distinctive sound. A curtain was then closed in front of the coins. More coins dropped, but the curtain remained closed. Participants could only hear but not see the second set of coins falling. Their task was to choose the total amount of coins on the floor, both the ones they saw and heard and the ones they only heard but did not see falling. The minimum number of coins displayed/heard was 1, the maximum was 5. The result range was from 3 to 7. In the **non-symbolic answer** version of this task (3 items), participants were presented with three images showing coins on the floor with an open curtain. Their task was to choose the image showing the total number of coins that are now on the floor. In the **symbolic answer** version of the task (3 items), participants were presented with an array of numerals from 0 to 9 in ascending order to choose from.

This task aimed to assess numerical processing at a crossmodal level, requiring a higher level of abstraction than unimodal tasks like the non-symbolic addition and subtraction tasks where only visual information is processed before answering the question. The addition of discrete sounds as stimuli adds a layer of abstraction that is not present in the other addition tasks (symbolic or non-symbolic) and ensures that responses must be based on a *truly abstract number sense, capable of representing any set of discrete elements* (Barth et al., 2003), independently from its physical nature and prior cultural learning of number symbols.

#### Symbolic arithmetic: addition and subtraction (S2)

In this task, participants had to solve traditional symbolic arithmetic problems in the range of 0–9, both addition (6 items) and subtractions (6 items), shown at the center of the screen. The answer format in this task was symbolic only, i.e., participants were presented with an array of numerals from 0 to 9 in ascending order below the problem to choose their answer from.

### Observation and interview sheets

To examine the usability of instructions and task presentation, test administrators collected information about participants' behavior during testing through semi-structured observation and interview sheets. Of special interest were the observations about the general use of the tablet and the tool's navigational features as well as participants' understanding of both video and verbal instructions and feedback elements in both groups.

The following questions (yes-no format) were answered for each participant and task: (1) Did the participant understand the purpose of the smiley? (2) Did the participant understand the use of the blue arrow as a navigational tool? To this aim, the test administrators asked the participants to describe the task, the role of the smiley, and the role of the arrow and evaluated that answer as a “Yes” or a “No.” These questions were followed by empty space for comments.

### Demographics and criterion validation tasks

After completion of the digitally administered tasks, all children received a paper notebook containing a demographic questionnaire as well as some control tasks. The questionnaire collected basic demographic data (age, gender, language spoken with mother). Control tasks were included to examine the criterion validity of the experimental tasks and were administered to both samples. The paper pencil control tasks were:

- ***TTR (Tempo Test Rekenen)*** (De Vos, 1992): a classical standardized measure of speeded arithmetic performance. Participants had 60 s for each subtest. Arithmetic difficulty increased systematically within each subtest list, with operands and results in the range of 1–100. As multiplication and division were not part of the participant's curriculum at that age, we used the addition and subtraction subtests only.
- **“*How many animals?”(Counting and transcoding)***: Since all of our experimental task assume basic counting skills, we included this self-developed counting task, in which ten paper sheets displaying a randomly arranged variable number of animals (range: 3–19) were presented successively to the participants, who reported how many animals they saw. Their oral answer was noted on a coding sheet by the test administrators. Furthermore, participants wrote down their answer on a separate coding sheet included in the participant notebook. This resulted in two separate measures: one for counting (oral) and one for transcoding ability (written).
- ***SYMP (Symbolic magnitude processing test)*** (Brankaer et al., 2017): a standardized measure of symbolic number comparison performance (1- and 2-digit, ranging from 1 to 10 and from 12 to 99, respectively). It includes a motor speed control task requiring participants to cross out the black shape in pairs of black/white shapes. Participants had 30 s for each subtest. Although number comparison abilities assessed by the SYMP test do not strictly constitute a measure of curricular learning goals, we choose to include it due to its well-recognized power to predict later differences in standardized mathematical tests and distinguish children with MLD from typically developing peers (see Schneider et al., 2017 for a meta-analysis). In contrast to the TTR scales and the counting task, correlation with the SYMP does not inform on the ability of our tasks to predict children's achievement on higher level learning goals but allows to compare performance in our tasks to another low-level predictor of later math competence.

### Design and procedure

#### Experimental design

To evaluate comprehensibility and effectiveness of the video instructions in comparison to classical verbal instructions, we implemented a between-group design in the two samples. All children solved the tasks on tablet computers, but under two different conditions. In the experimental condition (non-verbal condition), instructions were conveyed through a video of a person performing specific basic mathematical tasks, followed by a green smiley indicating successful solution of the task. Importantly, children did not receive any verbal instructions in the experimental condition. In the control condition (verbal condition), children received verbal instructions in German, the official instruction language for Mathematics in elementary schools in Luxembourg. Analogous to usual classroom conditions, test administrators read the instructions aloud to the children. In both conditions, tasks were presented visually on tablet computers, either through static images or animated “short stories.” In both samples, one group was allocated to the experimental non-verbal condition without language instructions and the other group was assigned to the verbal condition, respectively.

#### Task presentation

The three main tasks for Sample 1 were presented on iPads using a borderless browser window. Two children were tested simultaneously. They were connected to a local server through a secured wireless network set up by the research team at each school to store and retrieve data. The tasks were implemented using proprietary web-based assessment-building software under development by the Luxembourg Centre for Educational Testing. Sample 2 worked on Chromebooks instead of iPads. The advantage of Chromebooks is that they are relatively inexpensive, are optimized for web applications, and provide both touchscreen interactivity and a physical keyboard when necessary. Four children were tested simultaneously to speed up data collection.

After the initial setup of the hardware (server, wireless connection), participants were called into the test room in groups of two (Sample 1) or four (Sample 2) and seated individually on opposite sides of the room, allowing to run multiple test sessions simultaneously. Participants were randomly assigned to one of two groups. A trained test administrator supervised each participant during the test session. Since the tasks for Sample 2 used audio material, participants were provided with headphones, which they wore during the video instructions and the tasks.

Both samples were presented with either non-verbal or verbal instructions. In the non-verbal condition (experimental group), each participant was shown three items, with the exception of the comparison task, where ten instruction items were given to account for the less salient nature of the implicit “Where is more?” instruction. The video also clarified how to proceed to the next item by the person touching a blue arrow pointing rightwards on the top right corner of the screen, after which a new item was loaded. In the verbal condition (control group), the test administrator read the standardized oral instructions to the participant in German, thus mimicking traditional teaching and test situations. The instruction was repeated by the test administrator while the first practice item was displayed to facilitate the hands-on understanding of the task. After the instruction, participants were given three practice items with the same smiley-type feedback they had just witnessed (a happy green face for correct answers, an unhappy red face for wrong answers). After successful completion of the three practice items, the application moved on to the test items. If one or more answers were wrong, all three practice items were repeated once, including those that had been solved correctly in the first trial. At the end of this second run, the application moved on to the test items, even if one or more practice items had still been answered incorrectly. After each practice session, an animation showing a traffic light switching from red to green was displayed to notify children that the test was about to start.

At the end of the three tasks, a smiley face was displayed thanking the participants for their efforts. At the end of the individual testing sessions, all participants were regrouped in their classroom to complete the pen-and-paper measures instructed orally by the test administrators.

#### Scoring

Scores from symbolic and non-symbolic subgroups of items in most *experimental tasks* were averaged and operationalized as POMP (percentage of maximum performance) scores (Cohen et al., 1999), giving rise to two scores in each task. The exception was the symbolic arithmetic task in Sample 2, which by its nature included only symbolic answer formats, but offered both addition and subtraction items, producing one score for each operation type. All scores from the *criterion validation tasks* are expressed as POMP scores.

## Results

In line with our research questions outlined in the introduction, we will first report findings on participants' difficulties by experimental condition, as usability represents an important prerequisite. Results on the directly assessed difficulties will focus on understanding of feedback and navigation, whereas indirectly assessed difficulties comprise findings on repeated practice. This is followed by descriptive analyses including scale quality, tests of normality, and scale intercorrelations. As we also examined the convergent validity of our tasks (another prerequisite), which were based on existing measures, we subsequently report findings on the correlations with the external measures, i.e., the paper pencil tests (see Materials section). Finally, we will compare performance by experimental condition.

### Observation data

#### Directly assessed difficulties: understanding of feedback and navigation

The following results are based on the observation sheets for each task. Table 2 shows the number of participants that understood the smiley as a feedback symbol and the number of participants that understood the arrow as a navigational interface element. Discrepancies in the total number of participants are due to missing data points for some participants.

**Table 2:** Directly assessed difficulties by experimental condition.

**Sample**	**Task type**	**Condition**	**Smiley**	**χ^2^**	***df***	***p***	**Navigation**	**χ^2^**	***df***	***p***
1	Quantity correspondence	Verbal	46/46	1.01	1	0.315	45/46	6.03	1	0.014
		Non-verbal	45/46				38/46			
	Quantity comparison	Verbal	46/46	1.01	1	0.315	46/46	1.01	1	0.315
		Non-verbal	45/46				45/46			
	Ordering	Verbal	46/46	1.03	1	0.309	46/46	1.06	1	0.304
		Non-verbal	44/45				43/44			
2	Non-symbolic addition	Verbal	70/70	3.02	1	0.082	69/70	5.82	1	0.016
		Non-verbal	68/71				62/70			
	Non-symbolic subtraction	Verbal	70/70	n.a.			68/69	1.02	1	0.312
		Non-verbal	71/71				70/70			
	Cross-modal addition	Verbal	70/70	1.02	1	0.312	68/69	1.04	1	0.309
		Non-verbal	71/71				70/70			
	Symbolic arithmetic	Verbal	69/69	n.a.			68/68	n.a.		
		Non-verbal	71/71				71/71			

*n.a., not applicable due to 1-level factor*.

Summarily, we observed that all but a few participants had correctly understood the feedback symbols and the navigation arrow from the start.

#### Indirectly assessed difficulties: practice repetition

**Figure 2:**
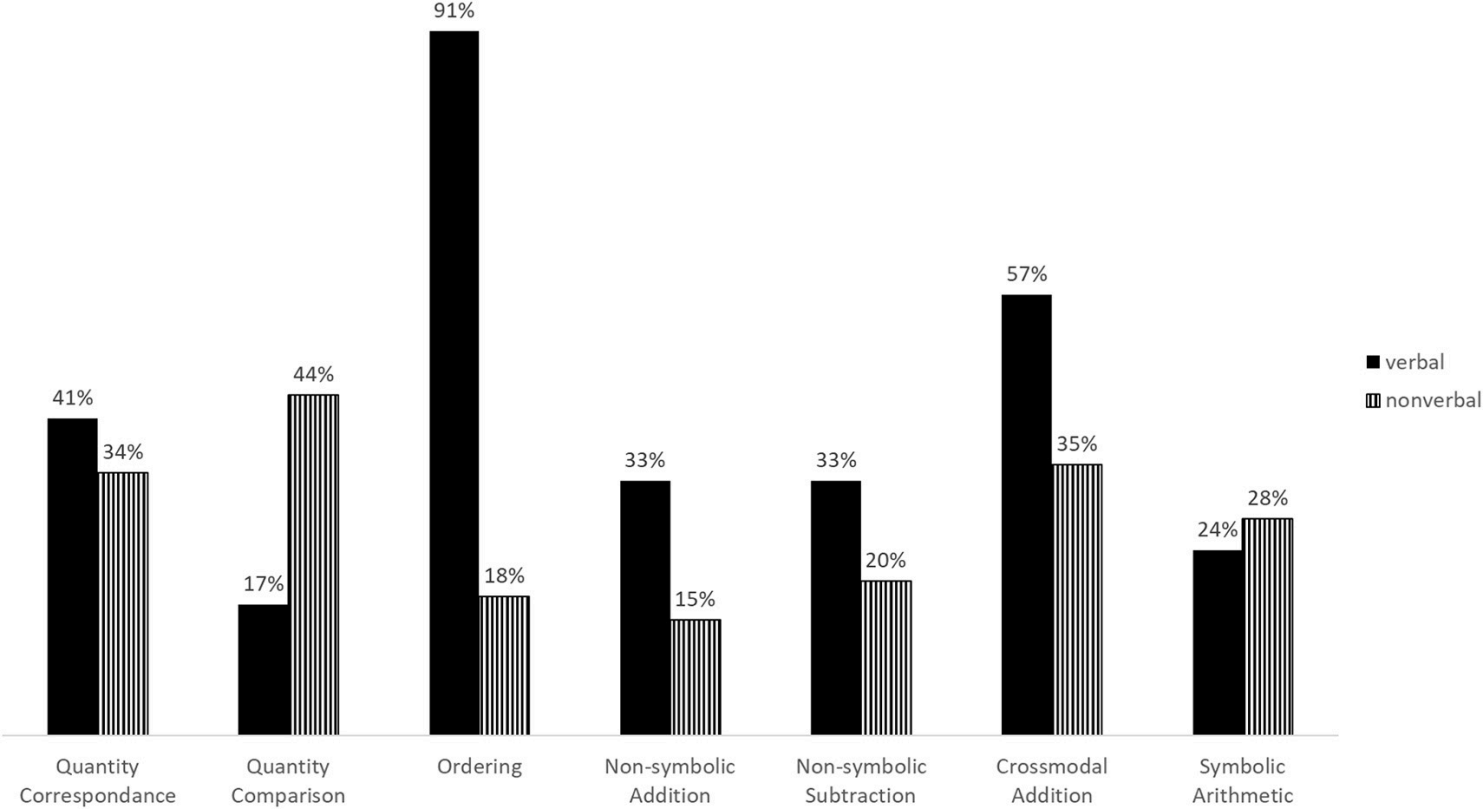
Percentage of repeaters by task and experimental group.

As an indirect measure of usability, we examined whether the number of participants that repeated the practice session of each task differed by experimental condition. Table 3 presents contingency tables and χ^2^-tests of association. Figure 2 presents percentage of repeaters per condition and task.

**Table 3:** Indirectly assessed difficulties (practice repetition) by experimental condition.

**Sample**	**Task type**	**Condition**	**Repeater**		**χ^2^**	***df***	***p***
			**No**	**Yes**			
1	Quantity correspondence	Verbal	27	19	0.55	1	0.46
		Non-verbal	33	17			
	Quantity comparison	Verbal	38	8	7.90	1	0.005
		Non-verbal	28	22			
	Ordering	Verbal	4	42	51.70	1	<0.001
		Non-verbal	41	9			
2	Non-symbolic addition	Verbal	47	23	5.81	1	0.016
		Non-verbal	60	11			
	Non-symbolic subtraction	Verbal	47	23	3.14	1	0.076
		Non-verbal	57	14			
	Cross-modal addition	Verbal	30	40	6.82	1	0.009
		Non-verbal	46	25			
	Symbolic arithmetic	Verbal	53	17	0.27	1	0.6
		Non-verbal	51	20			

The number of participants that repeated the practice session did not vary significantly between conditions in the *Quantity correspondence task*, the *Non-symbolic subtraction task* and the *Symbolic arithmetic task*. Fewer participants repeated the practice session in the non-verbal condition of the *Ordering, Non-symbolic addition* and *Cross-modal addition* tasks. Inversely, more participants repeated the practice session in the non-verbal condition of the quantity comparison task.

### Task descriptives

#### Internal consistency

Internal consistency of the experimental tasks in the first sample ranged from good to questionable (see Table 4). Only the *Ordering* task with non-symbolic answers showed unacceptable internal consistency. Due to the low number of items in each task, we estimated internal consistency without differentiation as to answer format in the second sample. While the *Symbolic arithmetic* task provided acceptable (Subtraction) to good (Addition) internal consistency, the three other tasks only reached poor to questionable consistency.

**Table 4:** Task performance, descriptives and non-verbal vs. verbal comparison.

**Task type**	**Cond**.	***N***	***M(POMP)* (*SD*)**	**Range**		**Internal consistency**		**Skewness**	**S-W**		**ANOVA (K-W)**	
				**Theor**.	**Emp**.	α	ω		***W***	***p***	χ^2^	***P***
**SAMPLE 1**												
Quantity correspondence (NS)	Verbal	46	0.86 (0.19)	0–1	0.08–1	0.813	0.852	−1.77	*0.69*	<0.001	1.47	>0.05
	Non-verbal	50	0.91 (0.16)	0–1	0.17–1			−2.54				
Quantity correspondence (S)	Verbal	46	0.96 (0.09)	0–1	0.63–1	0.746	0.819	−2.39	0.48	<0.001	1.63	>0.05
	Non-verbal	50	0.92 (0.17)	0–1	0.25–1			−2.63				
Quantity comparison (NS)	Verbal	46	0.95 (0.13)	0–1	0.50–1	0.892	0.899	−2.94	0.48	<0.001	1.62	>0.05
	Non-verbal	50	0.87 (0.27)	0–1	0–1			−1.94				
Quantity comparison (S)	Verbal	46	0.96 (0.14)	0–1	0.25–1	0.697	0.737	−3.54	0.48	<0.001	3.70	>0.05
	Non-verbal	50	0.88 (0.24)	0–1	0–1			−2.00				
Ordering (NS)	Verbal	46	0.78 (0.26)	0–1	0.25–1	0.462	0.521	−0.68	0.83	<0.001	0.60	>0.05
	Non-verbal	50	0.74 (0.25)	0–1	0–1			−0.80				
Ordering (S)	Verbal	46	0.92 (0.22)	0–1	0–1	0.735	0.763	−2.78	0.53	<0.001	3.02	>0.05
	Non-verbal	50	0.88 (0.23)	0–1	0–1			−2.08				
**SAMPLE 2**												
Non-symbolic addition (NS)	Verbal	46	0.78 (0.24)	0–1	0–1	0.495	0.553	−0.82	0.75	<0.001	1.94	>0.05
	Non-verbal	50	0.84 (0.19)	0–1	0.33–1			−0.69				
Non-symbolic addition (S)	Verbal	46	0.66 (0.31)	0–1	0–1			−0.40	0.84	<0.001	0.15	>0.05
	Non-verbal	50	0.67 (0.32)	0–1	0–1			−0.63				
Non-symbolic subtraction (NS)	Verbal	46	0.88 (0.21)	0–1	0–1	0.593	0.618	−1.88	0.61	<0.001	0.00	>0.05
	Non-verbal	50	0.89 (0.19)	0–1	0.33–1			−1.51				
Non-symbolic subtraction (S)	Verbal	46	0.62 (0.35)	0–1	0–1			−0.50	0.83	<0.001	1.27	>0.05
	Non-verbal	50	0.69 (0.33)	0–1	0–1			−0.78				
Cross-modal addition (NS)	Verbal	46	0.79 (0.26)	0–1	0–1	0.439	0.480	−0.90	0.75	<0.001	0.03	>0.05
	Non-verbal	50	0.79 (0.27)	0–1	0–1			−1.07				
Cross-modal addition (S)	Verbal	46	0.62 (0.33)	0–1	0–1			−0.49	0.86	<0.001	1.37	>0.05
	Non-verbal	50	0.57 (0.31)	0–1	0–1			−0.28				
Symbolic arithmetic (Add.)	Verbal	46	0.95 (0.14)	0–1	0–1	0.880	0.888	−4.76	0.32	<0.001	0.07	>0.05
	Non-verbal	50	0.94 (0.21)	0–1	0–1			−4.05				
Symbolic arithmetic (Sub.)	Verbal	46	0.85 (0.23)	0–1	0–1	0.787	0.803	−2.04	0.66	<0.001	0.01	>0.05
	Non-verbal	50	0.83 (0.27)	0–1	0–1			−1.84				

*POMP, Percentage of maximum performance; S-W, Shapiro-Wilk test of normality; K-W, Kruskal-Wallis ANOVA on ranks*.

#### Tests for normality

All task scores showed ceiling effects (somewhat less pronounced in Sample 2), independently from experimental group or the symbolic nature of the task, thus deviating significantly from the normal distribution (statistical tests for all subtests are reported in Table 4). Skewed distributions were expected considering the test was designed to differentiate at the bottom end of the ability distribution. Consequently, the Shapiro-Wilks tests showed substantial non-normality. Therefore, we conducted non-parametric analysis of variance to examine possible group differences in task performance.

#### Scale intercorrelations

In Sample 1 performances on almost all experimental tasks correlated significantly among each other (see Table 5). The exception was the *Quantity comparison* task (symbolic format), which did not correlate significantly with the *Quantity correspondence* task (non-symbolic format) and with the *Ordering* task (both formats).

**Table 5:** Scale intercorrelations: Sample 1.

**Scale intercorrelations**		**Quantity correspondence**	**Quantity comparison**	**Quantity comparison**	**Ordering**	**Ordering**
**Sample 1**		**(S)**	**(NS)**	**(S)**	**(NS)**	**(S)**
Quantity correspondence (NS)	Rho	0.516	0.424	0.189	0.436	0.296
	*p*	<0.001	<0.001	0.065	<0.001	0.003
Quantity correspondence (S)	Rho		0.374	0.212	0.294	0.215
	*p*		<0.001	0.038	0.004	0.036
Quantity comparison (NS)	Rho			0.612	0.443	0.381
	*p*			<0.001	<0.001	<0.001
Quantity comparison (S)	Rho				0.125	0.147
	*p*				0.225	0.153
Ordering (NS)	Rho					0.519
	*p*					<0.001

*S, Symbolic answer format; NS, Non-symbolic answer format; Rho, Spearman's rho*.

The reported correlations in the following paragraph are all significant (see Table 6). Letters in parentheses indicate the answer format (NS = non-symbolic; (S) = symbolic). In Sample 2, performances in *Symbolic arithmetic* (addition and subtraction) correlated with each other and with performance in all other tasks having a symbolic response format (i.e. *Non-symbolic addition, Non-symbolic subtraction*, and *Cross-modal addition*). Performance in *Symbolic arithmetic* did not correlate with performance in tasks requiring non-symbolic output, except for the *Non-symbolic subtraction* task. Performances in *Non-symbolic addition* and *subtraction (S)* correlated with performance on all other tasks. Performances in the two *Non-symbolic arithmetic (NS)* did not correlate with each other. Performance in *Cross-modal addition (S)* correlated with performance in all other tasks, except *Non-symbolic arithmetic* (i.e., *Non-symbolic addition* and *Non-symbolic subtraction*) with non-symbolic response formats. Performance in *Cross-modal addition (NS)* correlated with performance in all other tasks, except *Symbolic arithmetic*.

**Table 6:** Scale intercorrelations: Sample 2.

**Scale intercorrelations**		**NS Add**.	**NS Sub**.	**NS Sub**.	**Cross. Add**.	**Cross. Add**.	**Sym. Arith**.	**Sym. Arith**.
**Sample 2**		**(S)**	**(NS)**	**(S)**	**(NS)**	**(S)**	**(Add)**	**(Sub)**
Non-symbolic addition (NS)	Rho	0.257	0.157	0.317	0.306	0.162	0.138	0.046
	*p*	0.002	0.063	<0.001	<0.001	0.056	0.102	0.590
Non-symbolic addition (S)	Rho		0.335	0.372	0.244	0.260	0.236	0.417
	*p*		<0.001	<0.001	0.003	0.002	0.005	<0.001
Non-symbolic subtraction (NS)	Rho			0.342	0.197	0.042	0.241	0.231
	*p*			<0.001	0.019	0.619	0.004	0.006
Non-symbolic subtraction (S)	Rho				0.249	0.290	0.193	0.352
	*p*				0.003	<0.001	0.022	<0.001
Cross-modal addition (NS)	Rho					0.301	0.091	0.165
	*p*					<0.001	0.282	0.051
Cross-modal addition (S)	Rho						0.207	0.211
	*p*						0.014	0.012
Symbolic arithmetic (addition & subtraction) (S)	Rho							0.262
	*p*							0.002

*(S), Symbolic answer format; (NS), Non-symbolic answer format; Rho, Spearman's rho*.

### Criterion validity

In Sample 1, average performance (all experimental tasks combined) correlated significantly with all criterion validity tasks (see Table 7) except with the two-digit SYMP test.

**Table 7:** Criterion validity.

**Criterion validity**		**TTR+**	**TTR−**	**Counting**	**Counting**	**SYMP**	**SYMP**
				**(oral)**	**(written)**	**(one digit)**	**(two digit)**
**SAMPLE 1**							
Average test score (all tasks)	Rho	0.453	0.349	0.279	0.475	0.308	0.111
	*p*	<0.001	<0.001	0.006	<0.001	0.002	0.28
**SAMPLE 2**							
Average test score (all tasks)	Rho	0.431	0.355	0.441	0.499	0.409	0.26
	*p*	<0.001	<0.001	<0.001	<0.001	<0.001	0.002

*Rho, Spearman's rho*.

In Sample 2, average performance (all experimental tasks combined) correlated significantly with all criterion validity tasks.

### Comparison of task performance: verbal vs. non-verbal instructions

Analyses of variance (Kruskal-Wallis) on task scores with experimental group (verbal vs. non-verbal) as between-subjects factor revealed no significant differences in any of the tasks, neither in Sample 1 nor in Sample 2 (see Table 4). Overall performances were very high in the non-verbal and in the verbal condition (ranging between 57 and 96%), indicating that children succeeded comparably well in both conditions.

## Discussion

The purpose of the present study was to explore the possibility of measuring basic math competence in young children without using verbal instructions. To this aim we developed a series of computerized tasks presented on tablet-computers either verbally, using traditional language instructions or non-verbally, using video instructions repeatedly showing successful task completion and assessed whether the instruction type influenced task performance.

### Usability aspects

To check whether this new mode of instruction was effective, we assessed the comprehensibility of the tasks both directly and indirectly. Regarding the prior, the feedback symbols (the green happy and the red sad smiley faces during the instruction and practice phase) were easily understood by most if not all participants. The same is true for the navigation symbol (the arrow to both save the answer and switch to the next item).

As an indirect assessment of task comprehension, we examined differences in the number of participants that repeated the practice session of each task. Given the low difficulty level of the tasks presented during instruction and practice, we assumed that children who did not get the practice items right in their first attempt had not understood the purpose of the task at first and therefore needed a second run. In three tasks [*Quantity correspondence (S1), Non-symbolic subtraction (S2)*, and *Symbolic arithmetic (S2)*], the number of repeaters did not vary significantly, suggesting that non-verbal instructions can be understood as well as verbal ones. On the other hand, we observed significantly less repeaters in three other tasks [*Non-symbolic addition (S2), Ordering (S1)* and *Cross-modal addition (S2)*] when children were instructed non-verbally, implying that non-verbal instructions can be more effective than verbal ones in these situations. This tendency was especially pronounced in the *Ordering* task. Finally, we found an inverse difference in repeaters in the *Quantity comparison* task. Significantly more participants repeated the practice session of the *Quantity comparison* task when they received non-verbal instructions. Conveying “choose the side that has more” through a video showing successful task completion repeatedly seems to have worked less well than simply giving the participants an explicit verbal instruction to do so, even though we displayed more repetitions in this task than in the other tasks. This shows that not every task instruction can be easily replaced by non-verbal videos without adding unnecessary complexity. This result stands in stark contrast with our observations concerning the *Ordering* task, which was understood much better following non-verbal instructions. Because the verbal instruction requested to order items from left to right, the extreme difference in repeaters (91% vs. 18%) could possibly be attributed to the fact that reliable left /right distinction has not been achieved by children of this age. Notwithstanding, this observation illustrates well that a single word in the instruction can lead to a complete failure to understand the task at hand and that this can be easily avoided by using non-verbal video instructions. Taken together, our results based on the repetition of practice items suggest that non-verbal instructions are an efficient alternative to the classically used verbal instructions and might in some cases even be more direct and effective. However, they do not provide a universally applicable solution, because on rare occasions they fail to convey task instructions as clearly and unequivocally as spoken language.

Anecdotally, it appeared that children were generally highly motivated to complete our tasks and many asked if they could do them again. This might be due to the video-game-like appearance of the assessment tool, which differs considerably from the paper-and-pencil material that they encounter in everyday math classes, which probably helped to promote task compliance and motivation (Lumsden et al., 2016).

### Validity aspects

Scale intercorrelations indicate that performance in the three tasks assessed in Sample 1 (i.e., *Quantity correspondence, Quantity comparison, Ordering*) largely correlated, which may reflect the fact that they rely, at least in part, on the same basic numerical competences. While performance on the non-symbolic version of the *Quantity comparison* task did correlate with performance on most other experimental tasks, performance on the symbolic version of the *Quantity comparison task* shows less consistent correlations with performance on other tasks. Most strikingly, the latter does not correlate significantly with performance on the *Ordering* task, both symbolic and non-symbolic versions. This stands in contrast with most findings in recent literature that report strong correlation between performance on tasks measuring cardinality (*Quantity comparison* task) and ordinality (*Ordering* task) (e.g., Lyons et al., 2014; Sasanguie et al., 2017; Sasanguie and Vos, 2018). This might be due to reporting correlations for the whole sample without distinguishing instruction type: a large proportion of participants in the video condition of the task did not seem to correctly understand its purpose, which could explain the absence of correlation between its performance and any other task. Accordingly, the *Quantity comparison* task will need to be adapted in future studies. Sample 2 consisted of calculation tasks that were either presented in classical symbolic or more unusual non-symbolic and/or cross-modal format (i.e., *Symbolic addition and subtraction, Non-symbolic addition and subtraction, Cross-modal addition*). In this sample, performance in symbolic arithmetic correlated with performance in those tasks having a symbolic response format, but not those requiring non-symbolic answers. This points toward a special role of number symbol processing, in line with the importance of this ability for mathematics (e.g., Bugden and Ansari, 2011; Bugden et al., 2012). Interestingly, and in line with the importance of number symbols, performance in non-symbolic arithmetic tasks with symbolic output formats also correlated with all calculation tasks of Sample 2. While validating the main expectations concerning our task and their properties, conclusions concerning scale intercorrelations remain provisional at this stage, since all tasks could not be correlated with each other in the present design due to two different participant samples.

Considering the overall medium reliability of our experimental tasks, special care should be taken to include more items assessing performance in the different tasks in further developments of this project.

Finally, we observed that average performance of all experimental tasks combined correlated significantly with performance in most (Sample 1) to all (Sample 2) control tasks. The control tasks were chosen to cover the most established measures of basic math competences in young children, known to predict latter differences in standardized mathematical tests and distinguish children with MLD from typically developing peers. We therefore included tasks assessing children's abilities to count (Goldman et al., 1988; Geary et al., 1999; Passolunghi and Siegel, 2004; Willburger et al., 2008; Hornung et al., 2014), to compare symbolic magnitudes (De Smedt et al., 2009, 2013; Brankaer et al., 2017) and to calculate (De Vos, 1992; Geary et al., 1993; Klein and Bisanz, 2000; Locuniak and Jordan, 2008; Geary, 2010). The non-significant correlation between performance of the tasks in the first sample with performance in the two-digit symbolic number comparison task can be attributed to participant's lack of knowledge on two-digit numbers at the time of data collection (approx. 5 weeks of schooling) (MENFP, 2011; Martin et al., 2013).

### Task performance compared by experimental group

Type of instruction prior to the test did not affect participants' performance in any of the experimental tasks. We observed high average performance in both samples and similar performances in both experimental conditions. This leads us to conclude that instruction type does not seem have an observable effect on future task performance. In other words, explicit verbal instructions can be replaced by videos showing successful task completion for children to understand the functioning and purpose of the numerical and mathematical tasks. This is an important result when put in the context of multilingual settings in particular, where the language of instruction can have considerable negative effects on task performance. Indeed, video instructions seem to work as well as traditional verbal instructions while taking language out of the equation.

At this point, we want to stress that we do not claim that mathematics and language can be assessed independently (Dowker and Nuerk, 2016). Indeed, prior research has shown that while the logic and procedures of counting are stored independently from language, the learning of even small number words relies on linguistic skills (Wagner et al., 2015). Also, languages inverting the order of units and tens in number words negatively affect the learning of number concepts and arithmetic (Zuber et al., 2009; Göbel et al., 2014; Imbo et al., 2014). Other studies have highlighted that proficiency in the language of instruction (Abedi and Lord, 2001; Hickendorff, 2013; Paetsch et al., 2016; Saalbach et al., 2016) and, more specifically, the mastery of mathematical language are essential predictors of mathematics performance (Purpura and Reid, 2016). It also becomes increasingly clear that test language modulates the neuronal substrate of mathematical cognition (Salillas and Carreiras, 2014; Salillas et al., 2015; Van Rinsveld et al., 2017). On the other hand, we do claim that a testee's access to the assessment tools should not be limited by proficiency in a certain language. Although most existing tasks already use images to minimize linguistic load, they still rely on some form of verbal instruction or vocabulary that needs to be fully understood to solve the task correctly. We thus think that it is not sufficient to minimize language load in mathematics items, but that it would be preferential to remove linguistic demands altogether. Our results show that this can be achieved by using implicit video instructions that rely on participant's non-verbal cognitive skills.

### Limitations and future studies

A first limitation for the interpretation of our results are the medium internal consistency scores of many of our tasks. We aimed to explore as many tasks as possible using non-verbal instructions, while keeping total test time under 40 min due to children's limited attention span (Manly et al., 2001). This led to some psychometric compromises by offering only a few items per task and subscale (i.e., symbolic and non-symbolic answer format), especially for the tasks in the second sample. In the future, we will select the tasks with the highest potential of differentiating in the lower spectrum of ability and supplement them with more items.

To further differentiate experimental conditions, it would have been possible to present only word problems and exclude all animations in the verbal instruction group whenever possible. For example, instead of showing pigs moving into a barn, the animation could be replaced with a written/spoken story on pigs going into a barn before offering three possible answers. We expect that such a contrasted design would lead to more significant differences in task comprehension and would be particularly interesting to investigate differences in item functioning in relationship to the participant's language background. In order to provide a robust proof of concept for the valid use of video instructions we decided here to adapt a more conservative approach with minimal differences between the video and verbal conditions. However, it would be interesting to use also more contrasted conditions in future studies.

Additionally, we anecdotally observed that touchscreen responsiveness seemed to be an issue with more impulsive participants. Indeed, when the touchscreen did not react to a first touch by showing a bold border around the selected image, these participants switched to another answer. We speculate that they interpreted the non-response of the tool as a wrong answer on their part and choose to try another one. This is an unfortunate but important technical limitation that will be addressed in future versions of the application, as impulsivity and attention issues are strongly correlated with mathematical abilities, especially in the target population for this test (LeFevre et al., 2013). Finally, we want to stress the difference in participant's age between the two sets of tasks presented here. In future developments of this project, homogenous groups of children from the first half of the first grade should be targeted.

## Conclusion

Taken together, these preliminary results show that explicit verbal instructions do not seem to be required for assessing basic math competencies when replaced by instructional videos. While variations depending on the task and the quality of experimental instructions are present, video instructions seem to constitute a valid alternative to traditional verbal instructions. In addition, the video-game-like aspect of the present assessment tool was well received, contributing positively to children's task compliance and motivation. All in all, the results of this study provide an important and encouraging proof of concept for further developments of language neutral and fair tests without verbal instructions.

## Ethics statement

This study was carried out in accordance with the recommendations of the research ethics guidelines by the ethics review panel of the University of Luxembourg and has been approved by the former. All subjects gave written informed consent in accordance with the Declaration of Helsinki.

## Author contributions

MG and CS were responsible for the conception and design of the study. MG was responsible for the acquisition, analysis and interpretation of the data as well as the drafting of the paper. CH, TB, CM, RM, and CS made critical contributions to the interpretation of the data and the revision of the draft.

### Conflict of interest statement

The authors declare that the research was conducted in the absence of any commercial or financial relationships that could be construed as a potential conflict of interest.
